# Anticancer Activity of *Modified Tongyou Decoction* on Eca109 Esophageal Cancer Cell Invasion and Metastasis through Regulation of the Epithelial-Mesenchymal Transition Mediated by the HIF-1*α*-Snail Axis

**DOI:** 10.1155/2020/3053506

**Published:** 2020-09-29

**Authors:** Yongsen Jia, Xin Yan, Ying Cao, Wei Song, Guangji Zhang, Xueqin Hu

**Affiliations:** ^1^Chinese Medicine College, North China University of Science and Technology, Tangshan 063210, China; ^2^Postgraduate School, North China University of Science and Technology, Tangshan 063210, China; ^3^College of Basic Medical Science, Zhejiang Chinese Medical University, 548 Bin Wen Road, Hangzhou 310053, Zhejiang Province, China; ^4^The First Affiliated Hospital of Zhejiang Chinese Medical University, 310053 Hangzhou, Zhejiang, China; ^5^Center for Dynamical Biomarkers, Beth Israel Deaconess Medical Center, Harvard Medical School, 330 Brookline Avenue, Boston, MA 02215, USA

## Abstract

**Background:**

To explore the activity of *Modified Tongyou Decoction* (MTD) against Eca109 esophageal cancer (EC) cell invasion and metastasis and to ascertain the mechanism of its anticancer activity during the epithelial-mesenchymal transition (EMT) as mediated by the HIF-1*α*-Snail axis.

**Methods:**

Herbal compounds were prepared by ethanol extraction, and 6 herbs composing into MTD were dipped in water-free ethanol and filtered. The filtrate was collected and centrifuged. The remains were concentrated into a paste which was adjusted to 5000mg/mL concentration with DMSO. PBS was used to dilute the herbal solution to the half maximal inhibitory concentration. A hypoxic microenvironment was induced with CoCl_2_ in RPMI 1640 medium, in which Eca109 cells were cultured. The cytotoxicity of MTD was determined with CCK-8 assay. The activity of MTD against cell invasion and metastasis was explored with scratch assay and transwell assay. Western blot analysis was conducted to analyze the anticancer effects of MTD on the expression of HIF-1*α*-Snail axis- and EMT-related proteins. Quantitative RT-PCR was used to assess the mRNA expression of Snail. Immunofluorescence labeling was performed to examine how MTD affected the coexpression of Snail and HIF-1*α*.

**Results:**

The fifty percent inhibitory dose of MTD was 1410 *μ*g/mL in the normoxic environment and 1823 *μ*g/mL in the hypoxic environment based on the CCK-8 assay. The scratch assay showed that MTD significantly inhibited cell migration in both the normoxic and hypoxic microenvironments compared with the control groups (*P* < 0.05). The transwell assay showed that MTD significantly inhibited cell invasion in both the normoxic and hypoxic environments compared with the control groups (*P* < 0.05). Western blot showed that MTD significantly inhibited the expression of the HIF-1*α*, Snail, Vimentin, MMP-2, MMP-9, and VE-cadherin proteins and significantly induced the expression of E-cadherin in both the normoxic and hypoxic microenvironments compared with the control groups (*P* < 0.05). qRT-PCR indicated that MTD significantly inhibited Snail mRNA expression compared with that in the control groups (*P* < 0.05). Immunofluorescence assay showed that MTD significantly inhibited the coexpression of HIF-1*α* and Snail in both the normoxic and hypoxic microenvironments compared with the control groups (*P* < 0.05).

**Conclusion:**

MTD downregulated HIF-1*α*-Snail axis- and EMT-related proteins to inhibit EC cell invasion and metastasis in both the normoxic and hypoxic environments.

## 1. Background

Esophageal cancer (EC), a serious digestive tract cancer, is the 8th most common cancer and the 6th leading cause of cancer-related death, with a 5-year survival rate of only 15%–25% [1]. In China, the morbidity and mortality of EC rank 5th and 4th, respectively, among all cancers [2], and the situation has become very grim in recent years. Many researchers have discovered that the abnormal expression of transcription factors is closely correlated with malignant tumor onset and development, and among these transcription factors, Snail is a classic example that shows high levels of expression in several types of solid tumors [3–6]; Snail can induce epithelial-mesenchymal transition (EMT) [7], resulting in cancer cell invasion and metastasis and, consequently, leading to death.

Based on traditional Chinese medicine (TCM) theory, EC is characterized by different pathogeneses in early, middle, and late stages. Phlegm and Qi obstruct each other in the early stage, blood stasis pathogenesis emerges in the middle stage, and tiny Qi and deficient Yang are predominant in the late stage [8]. Clinically, by the time EC patients were diagnosed, 70 percent cases reached the middle-late stage, in which 67 percent cases were diagnosed as syndrome of blood stasis (SBS) [9]. Researchers presented that degree of blood stasis is closely related to the oxygen supply in tumor tissue and cells and that activating blood circulation and removing blood stasis improved hypoxia microenvironment of tumors [10, 11].

Li Dong-yuan, a famous medical practitioner, one of “Four TCM scholars in the Jin and Yuan Dynasty,” created an herbal formula named *Tongyou* Decoction (TD), which is a typical prescription for treating SBS. Based on the formula and TCM pharmacology theory, MTD was invented by our research team and patented in China [12]. A series of basic experiments and clinical trials were conducted showing that MTD inhibited Eca109 cell proliferation and downregulated the NF-*κ*B signaling pathway [13]. The present study explored the anticancer effects of MTD on the invasion and metastasis of Eca109 cells in the hypoxic microenvironment, aiming to elucidate the molecular mechanisms of the effects of MTD on EMT mediated by HIF-1*α*-Snail axis.

## 2. Methods

### 2.1. Materials and Reagents

MTD was made from the following components: *Marsdenia tenacissima* (*Marsdeniae tenacissimae* Caulis), 5 g; Barbed Skullcap Herb (*Scutellaria barbata* D. Don), 15 g; *Oldenlandia diffusa* (*Hedyotis diffusa*), 15 g; peach kernel (*Prunus persica* (L.) Batsch), 5 g; red flower (*Carthamus tinctorius* L.), 15 g; and rattletop (*Cimicifugae foetidae*), 10 g. These components were purchased from Beijing Tongrentang Pharmacy.

Eca109 cells were obtained from the Experiment Research Center of the Henan University of TCM.

RPMI 1640 medium was purchased from Corning Incorporated (USA). Fetal bovine serum (FBS), penicillin, streptomycin, and trypsin enzyme (0.25%) were purchased from Biological Industries (USA). CoCl_2_ was purchased from Sigma-Aldrich Inc (USA). The Cell Counting Kit-8 (CCK-8) was purchased from Zoman Biotechnology Co. Ltd. (China). Radio immunoprecipitation assay (RIPA) buffer was purchased from BestBio Company Ltd. (China). The bicinchoninic acid (BCA) protein quantitative assay kit was purchased from Multi Sciences Biotech Company (China). The HIF-1*α* mouse monoclonal antibody and the Snail mouse monoclonal antibody were purchased from Gene Tex Inc (USA). The VE-cadherin rabbit polyclonal antibody and the MMP-2 rabbit polyclonal antibody were purchased from Arigobio Laboratories Inc (China). The MMP-9 rabbit monoclonal antibody was purchased from Epitomics Inc (USA). Vimentin, E-cadherin, and GAPDH were purchased from Proteintech Group (USA). Goat anti-mouse IgG and anti-rabbit IgG were purchased from KPL Inc (USA). White polyvinylidene fluoride (PVDF) membrane was purchased from Roche Molecular Systems Inc (USA). ECL detection reagent and animal tissue/cell total RNA Kit were purchased from Zoman Biotechnology Co. Ltd. (China). Primers were designed by Sangon Biotech (Shanghai) Co. Ltd. (China). Sepharose, TRIzol reagent, and Taq DNA Polymerase were purchased from Invitrogen by Life Technologies (USA). SYBR Green qPCR Mix was purchased from Beyotime Biotechnology (China). Anti-mouse fluorescence IgG was purchased from Proteintech Group (USA).

### 2.2. Cell Culture

Eca109 cells were cultured in RPMI 1640 medium containing 10% FBS in an incubator at 37°C, 5% CO_2_, and saturated humidity.

### 2.3. MTD Preparation

The total dosage of the herbs is 65 g. To keep the weight of ethanol and compounds in the proportion 8 : 1, 520g water-free ethanol infused the herbs for 21 days and filtered. The filtrate was collected. Ethanol was added into the mixed herbs again for another 14 days and ethanol was collected, and both filtrates were combined and centrifuged at 3000 r·min^−1^ for 10 min, followed by evaporation of the ethanol. The remains were concentrated into a paste, which was dissolved in DMSO, adjusted to 13 mL, the storage concentration being modulated to 5000 mg/mL, which was calculated based on the crude herb dosage. The paste was stored at 4°C. PBS was added to dilute the herbal solution into different working concentrations.

### 2.4. CCK-8 Assay

The Eca109 cell suspension was prepared and adjusted to a concentration of 5 × 10^4^ cells/mL. The cells were seeded into a 96-well plate at 100 *μ*L/well and incubated in normoxic or hypoxic conditions for 24 h. The normoxic environment was 37°C with 5% CO_2_ and saturated humidity. The hypoxic environment was similar to the normoxic environment, but 10 mmol/L CoCl_2_ was added to the RPMI1640 medium, being 20% of the total volume. Six concentrations of MTD, diluted in RPMI 1640 medium as follows: 160 *μ*g/mL, 320 *μ*g/mL, 640 *μ*g/mL, 1280 *μ*g/mL, 2560 *μ*g/mL, and 5120 *μ*g/mL, were added at 100 *μ*L/well to triplicate wells. Two control groups were also prepared: a control group containing a complete culture medium (10% FBS) and a blank group containing serum-free culture medium. CCK-8 reagent was added 24 h later, and cells were cultured for 2 h in a 37°C incubator. The cells were analyzed in plates with an ELISA reader, and the optical density (OD) at 460 nm was measured. A graph was drawn with MTD concentration on the *x*-axis and the cell inhibition ratio on the *y*-axis, and the curve and formula were evaluated. According to the formula, inhibition ratio (%) = 1 − [(MTD group − blank group)/(control group − blank group)] × 100%, and the half maximal inhibitory concentration (IC50) was calculated for both the normoxic and hypoxic conditions.

### 2.5. Grouping and Treatment

There were 4 groups included in the study: the control group with oxygen (Group OC), the MTD group with oxygen (Group OM), the hypoxic control group (Group HC), and the hypoxic MTD group (Group HM). The cells in Group OC were cultured in complete RPMI 1640 medium 4 mL plus free RPMI 1640 medium 1 mL, the cells in Group OM were cultured in MTD IC50 (normoxic) solution adjusted by complete RPMI 1640 medium (total volume 5 mL), the cells in Group HC were cultured in complete RPMI 1640 medium 4 mL plus 10 mmol/L CoCl_2_ 1 mL, being 20% of the total volume, and the cells in Group HM were cultured in 10 mmol/L CoCl_2_ 1 mL, 20% of the total volume; MTD IC50 (hypoxic) solution was adjusted with complete RPMI 1640 medium, containing the CoCl_2_ volume.

### 2.6. Scratch Assay

Three horizontal lines were drawn across each well with a marker pen and ruler on a 6-well plate. The cells were seeded at a concentration of 5 × 10^5^ cells/well and were routinely cultured in complete medium for 24 h to allow for cell adherence. A 10 *μ*L pipette tip was used to scratch along the horizontal lines perpendicular to the back row. Each well was washed three times with PBS to remove the scratched cells and photographed immediately. The wells were grouped and treated with the different factors. The scratch width was recorded 24 h later and used in the following equation: cell migration distance (*μ*m) = scratch width at 0 h − scratch width at 24 h.

### 2.7. Transwell Experiment

Matrigel and free RPMI 1640 medium were mixed at a ratio of 1 : 6 and plated onto the bottom of the chamber and incubated at 37°C for 30 min. The cell concentration was adjusted to 1 × 10^5^ cells/mL, and 150 *μ*L cell solution was seeded on the upper layer of the transwell chamber and given 24 h to adhere. The cells were grouped according to the different treatments. The lower chamber was treated with medium containing 10% FBS and transferred to the incubator for 24 h. The medium was discarded, and PBS was added into the lower chamber to wash it thoroughly. The cells on the upper layer of the chamber were gently wiped off with a cotton swab. Four percent paraformaldehyde was added into the lower chamber to fix the cells and was then discarded. This step was followed by adding PBS to the chamber, and finally, crystal violet dye was added, and the cells were stained, fixed, and rinsed. Five fields of view in the center of the chamber were randomly selected under the microscope to calculate the number of infiltrating cells.

### 2.8. Western Blot

The cells were seeded in culture dishes at a concentration of 1 × 10^5^ cells/dish for 24 h to allow them to adhere to the bottom. The cells were then treated with different factors. After being treated, the cells were collected and RIPA buffer was added to extract total protein. The cells were centrifuged at 12000 r·min^−1^ and 4°C for 20 min. The supernatant was collected into EP tubes, and the protein concentration was determined with a BCA kit. Quantified protein was loaded, followed by heating for 5 min for protein denaturation. An 8% SDS-PAGE separating gel and a 10% stacking gel were prepared. After all the samples were loaded, electrophoresis was performed according to standard procedures. Electrotransfer was conducted for 8 h. The PVDF membrane was immersed in blocking buffer to block nonspecific proteins. Monoclonal and polyclonal antibodies were reacted with the membrane separately for 6 h (antibody concentrations were as follows: Snail, 1 : 2500; HIF-1*α*, 1 : 2000; MMP-2, 1 : 1000; MMP-9, 1 : 1000; VE-cadherin, 1 : 1000; E-cadherin, 1 : 1000; Vimentin, 1 : 1000; and GAPDH, 1 : 8000). The membrane was then incubated with IgG (antibody concentration 1 : 5000) for 1 h. ECL reagent was applied to develop the membrane. Gray values of protein were analyzed with Gel-Pro imaging. Relative OD value of target proteins = target proteins/GAPDH.

### 2.9. qRT-PCR

Total RNA was extracted with one-step TRIzol reagent, and RNA from each group was quantified with Q-5000 and used for reverse transcription. RT was carried out according to Table 1.

There were 2 steps included, RT and qPCR. The RT reaction conditions were 25°C for 15 min, 42°C for 30 min, then reverse transcriptase was inactivated at 85°C for 5 min, and finally, cDNA was prepared for qPCR. The qPCR reaction was carried out according to Table 2. The primer sequence for Snail and GAPDH is shown in Table 3.

The conditions for the 3-step PCR reaction were as follows: all reagents reacted at 94°C for 3 min; DNA denaturation at 95°C for 15 s, annealing at 60°C for 30 s, extending at 72°C for 30 s, 40 cycles; and finally extending at 72°C for 10 min. 3 *μ*L sample was added into 2% sepharose gel, and electrophoresis was performed for 90 min at 60 V. All factors' imaging was adapted with the Gel-Pro 4400 system, and OD value was determined with Gel-Pro Imaging. Relative OD value of target genes = target genes/GAPDH gene.

### 2.10. Immunofluorescent Labeling of Cells

The cells were divided into 4 groups and given different treatments for 24 h. The RPMI 1640 medium was discarded. Cell slides were prepared and rinsed twice with preheated PBS. Polyoxymethylene (concentration, 1 : 25) was added to fix cells for 30 min, followed by rinsing with PBS 3 times, blocking with 10% goat serum plus 0.1% Triton X-100 at room temperature, dehydrating, and adding the primary antibody buffer (concentration, 1 : 200). Cell slides were put into a humidified chamber at 4°C overnight, followed by rinsing with PBS, dehydrating, adding fluorescent IgG (concentration, 1 : 200), and incubating in the dark at 37°C for 24 h. The cells were rinsed with PBS 3 times, 4′,6-diamidino-2-phenylindole dihydrochloride (DAPI) was added, and slides were incubated and then washed with PBS. The slides were mounted with 30%∼50% glycerin. Finally, the slides were observed with a fluorescence microscope, and images were collected.

### 2.11. Statistical Analysis

The SPSS 22.0 software was used to analyze data. Gel-Pro imaging was used for the semiquantitative analysis of protein bands, mRNA, and fluorescent signals. Probit was used to calculate IC50 values based on the CCK-8 assay. The cell scratch widths, protein gray values, and mRNA values were expressed as (mean ± standard error). The data were tested for homogeneity of variance by one-way ANOVA, which was also applied for comparisons within multiple groups. Presuming that the data followed a normal distribution, the least significant difference (LSD) test was applied to compare differences in the data between each pair of groups. *P* < 0.05 was regarded as statistically significant.

## 3. Results

### 3.1. CCK-8 Assay

**Figure 1:**
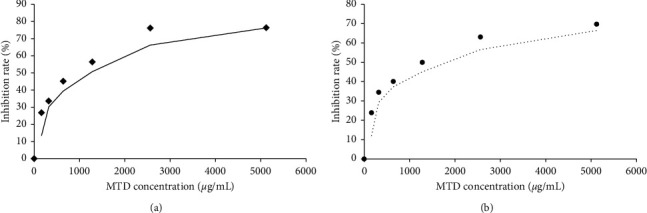
MTD inhibited Eca109 cells proliferation in a direct dose-effect manner in both normoxia and hypoxia. Notes: Relation between MTD concentration and cell inhibition rate showed the same tendency in both normoxia and hypoxia, fitted curve taking on cubic manner. (a) Dose-effect manner in normoxia: inhibition rate reached peak at 2560 *μ*g/mL, equal to the rate at 5120 *μ*g/mL, fitted formula, *Y*(%) = 9.738836 + 0.064047*x* + (−2 × 10^−5^)*x*^2^ + (1.93 × 10^−9^)*x*^3^. (b) Dose-effect manner in hypoxia, inhibition rate reached peak at 5120 *μ*g/mL, fitted formula, *Y*(%) = 9.255399 + 0.061135*x* + (−2.2 × 10^−5^)*x*^2^ + (2.48 × 10^−9^)*x*^3^.

As shown in Figure 1, MTD-mediated inhibition of Eca109 cell proliferation was measured with a CCK-8 assay under normoxic or hypoxic conditions. The *x*-axis indicates MTD concentration, and the *y*-axis shows the inhibition rate. A scatter plot and trend line were drawn, and the MTD IC_50_ was found to be ≈1410 *μ*g/mL in the presence of oxygen and 1823 *μ*g/mL in hypoxia.

### 3.2. Cell Scratch Assay

**Figure 2:**
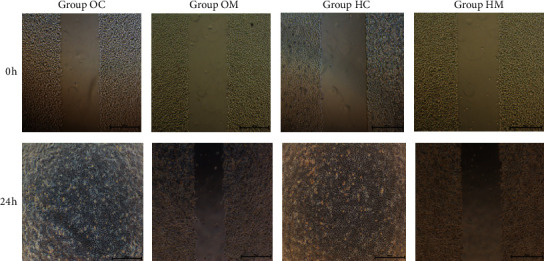
MTD inhibited Eca109 cell migration in both normoxia and hypoxia.

As shown in Figure 2 and Table 4, under different conditions, the ability of the cells to migrate was different. The cells in Group OC and Group HC reached confluency in the view field. It could be concluded that there was no difference in the migration ability of cells in normoxic and hypoxic environments. The cell migration in Group OM was inhibited, and the scratch width showed a significant difference compared with that in Group OC (*P* < 0.05). The cell migration in Group HM was also obviously inhibited, and the scratch width showed a significant difference from that in Group HC (*P* < 0.05).

### 3.3. Transwell Experiment

**Figure 3:**
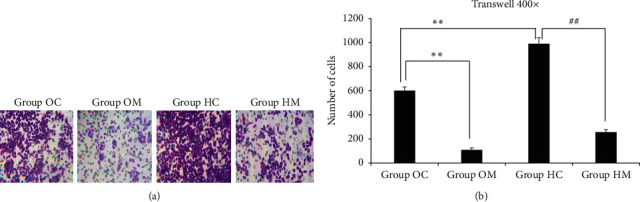
MTD inhibited Eca109 cell invasion in both normoxia and hypoxia. Note: (a) in Group OC, transwell cells were in vigorous proliferation; in Group OM, cells were infertile, showing light color; in Group HC, cells were in mass and proliferating exuberant; in Group HM, cells were inhibited greatly and mass disappeared. (b) In Group OM, cell number reduced remarkably, showing statistical difference from that in Group OC, ^*∗∗*^*P* < 0.05. What is noteworthy, in Group HC, cell number increased greatly, showing statistical difference from that in Group OC, ^*∗∗*^*P* < 0.05. In Group HM, cell number reduced sharply, showing statistical difference from that in Group HC, ^##^*P* < 0.05. Homogeneity of variance by one-way ANOVA; LSD test applied to compare statistical difference.

As shown in Figure 3, the number of cells in Group HC was significantly larger than that in Group OC (*P* < 0.05), suggesting the cell invasion ability of Group HC was increased by the hypoxic environment. The cell numbers in both the treatment groups were much smaller than those in the 2 control groups, and this difference was significant between groups within the same environmental conditions (*P* < 0.05), suggesting that MTD could inhibit cell invasion in both normoxic and hypoxic environments.

### 3.4. Western Blot

**Figure 4:**
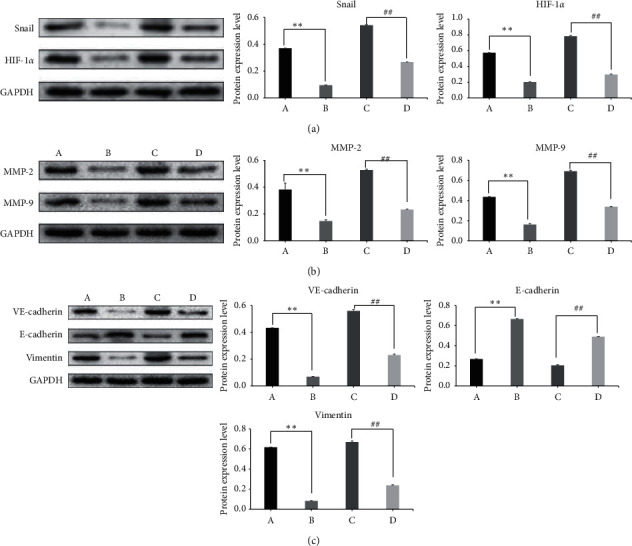
Protein expression of the HIF-1*α*-Snail axis and EMT-related proteins. Note*:* A, Group OC; B, Group OM; C, Group HC; D, Group HM. Compared with Group OC, ^*∗∗*^*P* < 0.05; compared with Group HC, ^##^*P* < 0.05.

As shown in Figure 4 and Table 5, the Snail and HIF-1*α* proteins were significantly overexpressed in Group HC compared with those in Group OC (*P* < 0.05). Protein expression of Snail and HIF-1*α* in Group OM and Group HM decreased significantly compared with that in Group OC and Group HC, respectively (*P* < 0.05). The expression of MMP-2 and MMP-9 showed the same trend as Snail and HIF-1*α* expression levels did in different groups.

In Group HC, VE-cadherin and Vimentin were overexpressed; however, E-cadherin expression was lower compared with that in Group OC. The protein expression of VE-cadherin and Vimentin in Group OM and Group HM was decreased; however, the E-cadherin protein was expressed at significantly higher levels than in Group OC and Group HC, respectively (*P* < 0.05).

### 3.5. qRT-PCR Assay

**Figure 5:**
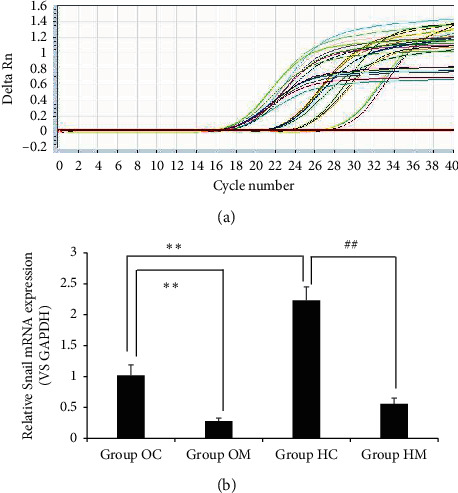
MTD inhibited Snail mRNA expression in both normoxia and hypoxia. Note: (a) amplification curve, as the target gene, Snail was measured with GAPDH. Value of Ct was in the domain of 15-30, and ▵Rn was in the domain of 0.5–1.5. (b) In Group OM, mRNA expression of Snail was inhibited remarkably, showing statistical difference from that in Group OC, ^*∗∗*^*P* < 0.05. What is noteworthy, in Group HC, Snail mRNA overexpressed greatly, showing statistical difference from that in Group OC, ^*∗∗*^*P* < 0.05. In Group HM, the mRNA expression was inhibited significantly, showing statistical difference from that in Group HC, ^##^*P* < 0.05. Homogeneity of variance by one-way ANOVA; LSD test applied to compare statistical difference.

As shown in Figure 5, transcription levels of Snail mRNA from the cells in Group HC significantly increased compared with those in Group OC (*P* < 0.05). The levels of Snail mRNA in Group OM and Group HM significantly decreased compared with those in Group OC and Group HC, respectively (*P* < 0.05). The results suggested that MTD inhibited Snail transcription both in normoxic and hypoxic microenvironments.

### 3.6. Immunofluorescent Labeling

**Figure 6:**
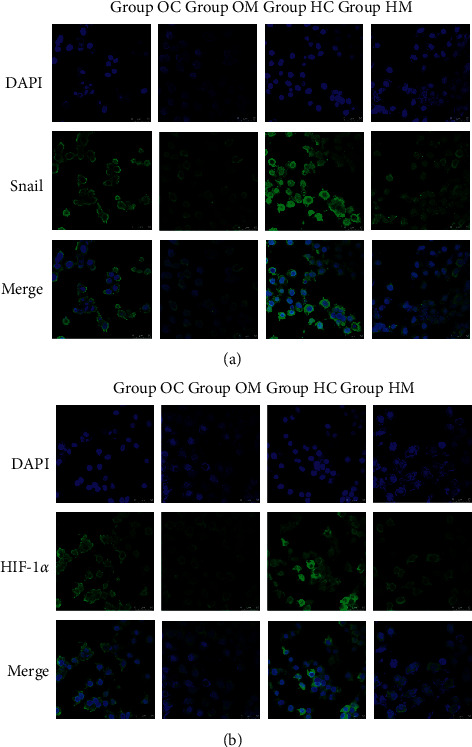
MTD inhibited Snail and HIF-1α fluorescent signals in both normoxia and hypoxia (200x). Note: blue fluorescence was DAPI, the green labeled Snail and HIF-1α, respectively. “Merge” was the combination of blue and green signals.

**Figure 7:**
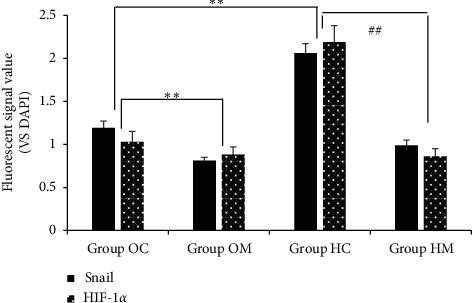
Relative OD values of HIF-1α and Snail fluorescent signals inhibited by MTD in both normoxia and hypoxia Note: in Group OM, HIF-1α and Snail fluorescent signals weakened remarkably, showing statistical difference from those in Group OC, ^*∗∗*^*P* < 0.05. What is noteworthy, in Group HC, both signals strengthened greatly, showing statistical difference from those in Group OC, ^*∗∗*^*P* < 0.05. In Group HM, both signals weakened significantly, showing statistical difference from those in Group HC, ^##^*P* < 0.05. Homogeneity of variance by one-way ANOVA; LSD test applied to compare statistical difference

The expressions of HIF-1*α* and Snail were observed with immunofluorescence. As shown in Figures 6 and 7, signals from HIF-1*α* and Snail are labeled in green. Both signals strengthened and localized to the cytoplasm in Group HC, and the signal intensities were stronger than those in Group OC (*P* < 0.05). The results suggested that HIF-1*α* and Snail were closely related in the hypoxic environment. In Group OM and Group HM, both the HIF-1*α* and Snail fluorescent signals were significantly lower than those in Group OC and Group HM, respectively (*P* < 0.05), indicating that MTD simultaneously suppressed HIF-1*α* and Snail.

## 4. Discussion

The microenvironment is an essential factor in cancer cell proliferation, development, and infiltration, and hypoxia plays an important role during these processes. HIF-1*α* activates transcription factors, such as Snail, twist, and ZEB1, under hypoxic conditions [14–16], promoting EMT pathogenesis and leading to cancer cell acquisition of stem cell activity. Conversely, EMT stimulates changes in the microenvironment.

EMT is a process by which epithelial cells lose their polarity and adhesion ability, gain migratory and invasive properties, and become mesenchymal stem cells [17]. After migrating and adhering to distal organs, tumor cells change their mesenchymal characteristics to epithelial characteristics again, fixing on target organs and proliferating without limit. Therefore, EMT onset is an obvious example of tumor invasion, infiltration, and metastasis [18]. A transcription factor characterized by a zinc finger structure, Snail, is one of the initial factors that induce EMT, typical pathobiological behavior for cancers [19]. Furthermore, as a cofactor for multiple signaling pathways involved in EMT, when Snail is highly expressed, it enhances the motility and invasiveness of tumor cells via downregulation of epithelial markers and upregulation of mesenchymal markers. Clinical reports have suggested that Snail overexpression is closely related to poor prognosis and high invasiveness of cancers [20].

Some studies have shown evidence that SBS is an important pathological factor for tumor onset [21–23], and the essence of blood stasis is an obstruction of oxygen supply [24]. Malignant tumors grow quickly, resulting in poor oxygen supply to tumor tissues and activating HIF-1*α* overexpression. Snail tends to be activated by HIF-1*α*, which has 2 special loci to bind Snail [14]. EMT is initiated by HIF-1*α*-Snail axis activation, causing tumor cell invasion and migration. Accordingly, it is assumed that SBS is connected to EMT via the HIF-1*α*-Snail axis.

Nationally patented MTD functions by detoxifying and activating blood stasis, thus treating the syndrome of blood stasis. MTD contains six components, *Marsdenia tenacissima, Scutellaria barbata* D. Don*, Hedyotis diffusa, Prunus persica* (L.) Batsch*, Carthamus tinctorius* L., *and Cimicifugae foetidae*, all of which are in common use for anticancer in TCM clinic. *Marsdenia tenacissima* (active constituent, Tenacissoside H) extract inhibits different cancer types including non-small cell lung cancer (NSCLC), malignant tumors, and hepatic carcinoma [25]. A new neo-clerodane diterpenoid, barbatin H, together with fifteen known analogues were isolated from *Scutellaria barbata* D.Don, and a study turned out that the series of neo-clerodane diterpenoids exhibited varying degrees of cytotoxic activities against the growth of tumor cell lines [26]. A chemical study was conducted on *Hedyotis diffusa* which investigated the cytotoxicity of the obtained compounds on eight tumor cell lines, showing that there were three iridoid glycosides of shecaoiridoidside A-C and a cerebroside of shecaocerenoside A. The cytotoxicity of all compounds against human tumor cell lines was evaluated, and new compound 3 exhibited evident cytotoxicity to all tumor cell lines [27]. *Prunus persica* (L.) Batsch extract was effective in relaxing serotonin (5-HT)- or angiotensin II-induced contraction; furthermore, the extract attenuated Ca^2+^-induced vasoconstriction by IP3 receptors in the SR membrane [28]. The findings imply that *Prunus persica* (L.) Batsch extract may be a natural anti-blood stasis agent. *Carthamus tinctorius* L. extract was applied to ischemia-reperfusion (I/R) brain injury in rats, showing that it can reduce cerebral infarction [29], which is associated with antioxidation property. A phytochemical study of *Cimicifugae foetidae* indicated that eight cycloartane-type triterpenes (1–8) were isolated and the new compounds showed moderate and broad-spectrum cytotoxicity against 5 human cancer cell lines [30]. MTD was prepared with ethanol extraction, which is the optimal way to dissolve various chemical components. The active ingredients from the compound are extracted fully and are of high quality.

The cytotoxicity of MTD was determined using a CCK-8 assay both in normoxic and hypoxic environments. Six concentrations were applied to ascertain the relation between dose and effect. The results showed that there was a dose-dependent effect; the relation between the MTD concentration and the inhibition rate was similar to the cubic formula. The IC50 lies between 1400 and 2000 *μ*g/mL, which suggests a satisfying anti-EC effect. Compared with one another, the IC50 in hypoxia was higher than that in normoxia, suggesting that the ability of Eca109 cells to proliferate is stronger in the hypoxic environment.

The scratch assay showed that the ability of Eca109 cells to migrate is no different between normoxic and hypoxic environments; however, the transwell experiment showed that the ability of the cells to invade became stronger in hypoxic environments than it did in normoxic environments. The results suggested that hypoxia offers a more favorable condition for Eca109 cell invasion. MTD inhibited cell invasion and migration in both normoxic and hypoxic environments.

Malignant tumors grow out of control, obstructing the oxygen supply, leading to HIF-1*α* overexpression, and inducing cancer cell EMT, during which E-cadherin is downregulated and Vimentin is upregulated [31]. By involving EMT, VE-cadherin, another member of the cadherin family, promotes the potency of cancer cell metastasis [32]. MMP-2 and MMP-9 degrade the extracellular matrix, playing an important role in cancer cell invasion, metastasis, and angiogenesis, which are impacted by EMT formation [33]. Western blot analysis showed that hypoxia activated the HIF-1*α*-Snail axis, through which EMT-related proteins, Vimentin, MMP-2, MMP-9, and VE-cadherin, were overexpressed, and E-cadherin expression was suppressed. MTD could inhibit the HIF-1*α*-Snail axis- and EMT-related proteins, except for E-cadherin, which was overexpressed. To evaluate the targets of the anticancer activity of MTD and, specifically, to determine whether it can affect gene expression, qRT-PCR was performed to quantify the differences in the Snail mRNA levels between normoxic and hypoxic environments. It is proven that MTD inhibited Snail gene transcription.

Immunofluorescent labeling was used to verify the relation between Snail and HIF-1*α*. The results showed that coexpression of Snail and HIF-1*α* was similar in the hypoxic microenvironment. MTD could modulate their coexpression regardless of the normoxic or hypoxic conditions.

## 5. Conclusion

In conclusion, the present study demonstrated that hypoxia-activated HIF-1*α*, initiating EMT mediated by Snail, improves Eca109 cell invasion and metastasis. The compound MTD downregulated EMT mediated by the HIF-1*α*-Snail axis, consequently inhibiting Eca109 cell invasion and metastasis.

## Figures and Tables

**Table 1:** RT system.

Reagent	Amount
5 × Hifair®II buffer	4 *μ*L
Hifair®II enzyme mix	2 *μ*L
Oligo (dT)_18_ (50 *μ*M)	1 *μ*L
Random primer N6 (50 *μ*M)	1 *μ*L
Total RNA	2 *μ*g
RNase-free ddH_2_O	20 *μ*L (final volume)

**Table 2:** qPCR system.

Reagent	Amount
BeyoFast™ SYBR Green qPCR MIX (2×)	10 *μ*L
Forward and reverse primer mix (3*μ*M each)	2 *μ*L
Template DNA	2 *μ*L
RNase-free water	20 *μ*L (final volume)

**Table 3:** Primer sequence.

Factor	Sequence
Snail	FW, 5′-GGGGTACCGGAAGCTGCTCT-3′
	RV, 5′-CCGCTCGAGGATTAGAGTCC-3′

GAPDH	FW, 5′-ACCACAGTCCATGCCATCAC-3′
	RV, 5′-TCCACCACCCTGTTGCTGTA-3′

**Table 4:** Regrowth degree of Eca109 cell was affected by different factors (*μ*m, $\bar{x}\pm s$).

	0 h	24 h
Group OC	661.44 ± 4.46	0.00 ± 0.00
Group OM	669.37 ± 3.75	478.59 ± 11.53^*∗∗*^
Group HC	681.57 ± 5.88	0.00 ± 0.00
Group HM	649.50 ± 9.87	607.45 ± 6.90^##^

Note: at 0 h time point, scratch width showed no statistical significance among 4 groups, one-way ANOVA. At 24 h time point, scratch width disappeared in Group OC and Group HC. In Group OM, scratch width was still present, showing statistical difference from that in Group OC, ^*∗∗*^*P* < 0.05. In Group HM, scratch width was still present, showing statistical difference from that in Group HC, ^##^*P* < 0.05. Homogeneity of variance by one-way ANOVA; LSD test applied to indicate statistical difference

**Table 5:** Relative gray values of protein band of the HIF-1*α*-Snail axis and EMT-related proteins ($\bar{x}$ *±* *s*).

	Snail	HIF-1ل	MMP-2	MMP-9	VE-cadherin	E-cadherin	Vimentin
Group OC	0.37 ± 0.0014	0.57 ± 0.0024	0.38 ± 0.0492	0.44 ± 0.0024	0.43 ± 0.0017	0.27 ± 0.0034	0.62 ± 0.0014
Group OM	0.09 ± 0.0026^*∗∗*^	0.20 ± 0.0033^*∗∗*^	0.15 ± 0.0097^*∗∗*^	0.16 ± 0.0103^*∗∗*^	0.07 ± 0.0023^*∗∗*^	0.66 ± 0.0058^*∗∗*^	0.08 ± 0.0023^*∗∗*^
Group HC	0.54 ± 0.0041^*∗∗*^	0.78 ± 0.0088^*∗∗*^	0.53 ± 0.0075^*∗∗*^	0.69 ± 0.0098^*∗∗*^	0.56 ± 0.0090	0.20 ± 0.0076	0.67 ± 0.0118
Group HM	0.27 ± 0.0012^##^	0.30 ± 0.0051^##^	0.23 ± 0.0040^##^	0.34 ± 0.0038^##^	0.23 ± 0.0074^##^	0.49 ± 0.0029^##^	0.24 ± 0.0059^##^

Note: in Group OM, except E-cadherin overexpression, the rest 6 proteins' expression was downregulated, showing statistical difference from that in Group OC, ^*∗∗*^*P* < 0.05. But in Group HC, Snail, HIF-1α, MMP-2, and MMP-9 was upregulated, showing statistical difference from that in Group OC, ^*∗∗*^*P* < 0.05. In Group HM, except E-cadherin overexpression, the rest 6 proteins' expression was downregulated, showing statistical difference from that in Group HC, ^##^*P* < 0.05. Homogeneity of variance by one-way ANOVA; LSD test applied to compare statistical difference.

## Data Availability

The data used to support the findings of this study are included within the article.

## Abbreviations

MTD: Modified Tongyou Decoction
EC: Esophageal cancer
EMT: Epithelial-mesenchymal transition
TCM: Traditional Chinese medicine
SBS: Syndrome of blood stasis
FBS: Fetal bovine serum
CCK-8: Cell counting kit-8
RIPA: Radio immunoprecipitation assay
BCA: Bicinchoninic acid
PVDF: Polyvinylidene fluoride
OD: Optical density
IC50: The half maximal inhibitory concentration
LSD: Least significant difference.
